# What influences life expectancy in people with dementia? Social support as an emerging protective factor

**DOI:** 10.1093/ageing/afae044

**Published:** 2024-03-15

**Authors:** Iris Blotenberg, Melanie Boekholt, Bernhard Michalowsky, Moritz Platen, Francisca S Rodriguez, Stefan Teipel, Wolfgang Hoffmann, Jochen René Thyrian

**Affiliations:** Deutsches Zentrum für Neurodegenerative Erkrankungen (DZNE), site Greifswald, Ellernholzstraße 1-2, 17489 Greifswald, Germany; Deutsches Zentrum für Neurodegenerative Erkrankungen (DZNE), site Greifswald, Ellernholzstraße 1-2, 17489 Greifswald, Germany; Deutsches Zentrum für Neurodegenerative Erkrankungen (DZNE), site Greifswald, Ellernholzstraße 1-2, 17489 Greifswald, Germany; Deutsches Zentrum für Neurodegenerative Erkrankungen (DZNE), site Greifswald, Ellernholzstraße 1-2, 17489 Greifswald, Germany; Deutsches Zentrum für Neurodegenerative Erkrankungen (DZNE), site Greifswald, Ellernholzstraße 1-2, 17489 Greifswald, Germany; Deutsches Zentrum für Neurodegenerative Erkrankungen (DZNE), site Rostock, Gehlsheimer Str. 20, 18147 Rostock, Germany; Department of Psychosomatic Medicine, University Hospital Rostock, Gehlsheimer Str. 20, 18147 Rostock, Germany; Deutsches Zentrum für Neurodegenerative Erkrankungen (DZNE), site Greifswald, Ellernholzstraße 1-2, 17489 Greifswald, Germany; Institute for Community Medicine, University Medicine Greifswald, Ellernholzstr. 1-2, 17489 Greifswald, Germany; Deutsches Zentrum für Neurodegenerative Erkrankungen (DZNE), site Greifswald, Ellernholzstraße 1-2, 17489 Greifswald, Germany; Institute for Community Medicine, University Medicine Greifswald, Ellernholzstr. 1-2, 17489 Greifswald, Germany

**Keywords:** dementia, risk factor, mortality, community, social environment, emotional support, primary care, longitudinal study, older people

## Abstract

**Background:**

The aim of this study was to investigate the role of support from the social environment for the life expectancy in people with dementia beyond well-established individual demographic and clinical predictors over a period of up to 8 years.

**Methods:**

The analyses are based on data from 500 community-dwelling individuals in Germany who tested positive for dementia and were followed up for up to 8 years. Life expectancy was examined in relation to perceived social support as well as well-established socio-demographic (age, sex) and clinical predictors (cognitive status, functional status, comorbidities), using Cox regressions.

**Results:**

Greater support from the social environment reduced the risk of mortality (hazard ratio [HR]: 0.78; 95% confidence interval [CI]: 0.63–0.98), with the role of emotional support being particularly important. Furthermore, higher age was associated with an increased mortality risk (HR: 1.08; 95% CI: 1.05–1.11), while female sex (HR: 0.64; 95% CI: 0.48–0.85) and higher cognitive (HR: 0.96; 95% CI: 0.93–0.98) and functional status (HR: 0.91; 95% CI: 0.86–0.97) were associated with higher life expectancy.

**Conclusion:**

Our study provides novel evidence that less support from the social environment, especially emotional support, is a risk factor for shorter life expectancy in people with dementia—beyond known clinical factors. Not only the clinical and caregiving needs but also their psychosocial needs of individuals with dementia should be emphasised.

## Key Points

- Support from the social environment predicted life expectancy in people with dementia beyond clinical factors.
- The dimension of emotional support played a particularly important role for life expectancy.
- Not only physical and healthcare needs but also psychosocial needs of people with dementia should be emphasised.

## Introduction

In many countries, the number of people with dementia (PwD) is rising; as of now, it exceeds 50 million and is anticipated to triple by the year 2050 [1, 2]. PwD have a reduced life expectancy compared to healthy individuals [3, 4]; however, there is considerable inter-individual variation [5, 6]. Well-researched is the role of individual patient factors, such as demographic (age, sex) and clinical factors (dementia severity, comorbidity burden); it has been shown that younger age, female sex, higher cognitive status, better functional status and lower comorbidity burden are associated with longer life expectancy [7, 8].

Social distancing measures due to the COVID-19 pandemic have brought into focus how crucial the social environment is for the well-being and health of PwD [9]. Several studies have demonstrated that the social environment plays a significant role in the progression of dementia symptoms, including neuropsychiatric symptoms, as well as in maintaining mental and physical health [10–13]. Additionally, social isolation has been identified as a key modifiable factor in dementia prevention [2, 14]. However, little attention has been paid to the potential role of the social environment in the life expectancy of PwD.

There are long-established links between the social environment, well-being and health [15–17]. Of particular interest is the functional dimension of the social environment, often referred to as social support [15, 18, 19]. Studies have shown that the functionality of the social environment—the ‘perception or experience that one is cared for, esteemed, and part of a mutually supportive social network’ [17]—is more strongly associated with health and well-being than formal or structural characteristics of the network [18, 20]. Different types of social support can be distinguished, such as emotional support, practical (or instrumental) support and social integration [15, 17, 21].

### The present study

Previous research on the life expectancy of PwD has largely overlooked the influence of the social environment. This could be due to the fact that the commonly used data sources, like registry data, claims data or data from memory clinics typically lack information on psychosocial factors. In this study, we utilise data from a sample of community-dwelling PwD in Germany, who were visited at home by specially qualified nurses, comprehensively interviewed and followed for up to 8 years. The objective of this study was to examine the impact of support from the social environment (both in total and in its subdimensions) on life expectancy of PwD over a period of up to 8 years.

## Methods

### Sample and study procedure

The present longitudinal study is based on data from the DelpHi-MV trial (dementia: life- and person-centred help in Mecklenburg-Western Pomerania, Germany) [22, 23]. Originally, the study had been conducted as a randomised controlled trial to examine the clinical effectiveness and cost-effectiveness of Dementia Care Management (DCM) compared to care as usual (CAU) after 1 and 2 years [23, 24]. DCM is a 6-month intervention in which specially trained nurses provide individualised support for the management and coordination of care for community-dwelling PwD. Further details can be found elsewhere [22]. After the effectiveness study had concluded, the sample was continued to be monitored in a prospective cohort study for a total of 8 years. The study received ethical approval from the ethics committee of the Chamber of Physicians of Mecklenburg-Western Pomerania—registry number: BB 20/11.

In total, 6,838 community-dwelling patients were assessed for eligibility across 125 participating primary care practices. Of these, 1,166 patients screened positive for dementia (DemTect score < 9) and met the additional inclusion criteria (age ≥ 70, living at home). Out of these, 634 patients provided their consent to participate in the study [25]. A few months after study enrollment (median: 3 months), the baseline assessment was conducted in participants’ homes. Specially trained nurses assessed cognitive performance and interviewed patients about their care situation, health status and psychosocial situation, among other factors. The analyses presented here are based on self-report data collected at baseline. Subsequently, participants were followed for up to 8 years. The baseline assessment was completed by 500 participants from 2012 to 2015. Figure S1 displays the complete participant flow.

### Measures

#### Primary outcome: mortality

The primary outcome of this study was mortality, defined as all-cause mortality in days since the baseline assessment over a time period of 8 years. If patients discontinued their participation in the study, for instance, due to death, relocation to a nursing home or withdrawal of consent, the exact date of such events was recorded. In addition, vital status was verified through inquiries with the local resident registration offices.

#### Predictors

Socio-demographic data (age, sex, living situation), clinical variables (cognitive and functional status), and social support were assessed at baseline through an extensive, standardised, computerised face-to-face interview at the participants’ homes by specially qualified nurses [22].

*Social Support* was assessed using the FSozU K-22 (‘Questionnaire for the assessment of social support’) [26], a measure of perceived social support from family, friends or acquaintances. The questionnaire has undergone extensive validation and has been widely used in the general population and in clinical studies [21, 26, 27]. It consists of 21 items, which assess 3 subdimensions of social support. The first dimension is emotional support, capturing the experience of closeness, trust, being liked, being accepted and receiving empathy (10 items). Example items include ‘There are people who accept me as I am’, ‘There are people who share both joy and sorrow with me’ and ‘I have friends/family who are good at listening when I need to talk’. The second dimension is practical support, the experience of help with everyday problems, e.g. ‘When I am sick, I can ask friends or family members to help me with important tasks (e.g. shopping) without hesitation.’ [5 items]). The third dimension is social integration, capturing a sense of belonging to a social network, e.g. ‘There is a community of people (circle of friends, group) to which I feel a sense of belonging.’ [7 items]). Each item is rated on a five-point Likert scale, where 1 represents ‘does not apply’ and 5 represents ‘exactly applicable.’ The mean of the items was calculated to obtain the total and the subscores.

The *cognitive status* was assessed using the Mini Mental State Examination (MMSE) [28], which yields scores from 0 to 30, with lower scores indicating greater cognitive impairment.

The *functional status* was assessed using the Bayer Activities of Daily Living scale (B-ADL) [29], which yields scores ranging from 1 to 10. It was recoded so that higher values indicate higher functional status.

Patients’ *comorbidities* at baseline were extracted from their medical records to calculate the Charlson comorbidity index. It ranges from 0 to 37, with higher values indicating a greater comorbidity burden [30].

*Group allocation* (DCM vs. CAU) was included as a covariate in all models because the study originally began as a randomised controlled trial.

### Statistical analysis

Data processing and all statistical analyses were carried out using R [31]. The significance level was set to *p* < 0.05. The Charlson comorbidity index [30] was calculated using the package *comorbidity* [32]. The number of missing values in the predictor variables was low. Missing values in the MMSE (6%), B-ADL (3%) and FSozU K-22 (6%) were addressed through multiple imputations using the package *mice*. Twenty imputed datasets with 50 iterations each were generated. As a sensitivity analysis, all analyses were also conducted using the original data without imputations. Cox regressions were computed using the package *survival* [33]. The results are reported as pooled estimates across the 20 multiple imputed datasets, with pooling conducted using the *hmisc* package [34].

Initially, we computed the influence of social support (overall) on life expectancy (Model 1.1). Subsequently, we included previously reported socio-demographic (age, sex) and clinical predictors (cognitive status, functional status, comorbidities) and covariates (living situation, group allocation) in the model and examined whether social support had an additional impact on life expectancy (Model 1.2). We repeated this process for the subdimensions of social support (Models 2.1–3.3).

## Results

### Descriptive statistics

Table 1 presents the descriptive statistics for the study participants. They had a mean age of 80.4 years (*SD* = 5.5), with a higher proportion of women (59%) than men. Half of the participants (50%) were living with a partner. Of the 500 participants, 228 had passed away within 8 years following the baseline assessment. Median survival time was 5.38 (95% confidence interval (CI) [4.95, 6.16]).

**Table 1:** Description of participant characteristics

Variable	Range	*M* (*SD*), *n* (%)
Age in years, M (SD)		80.4 (5.5)
Sex		
Female, *n* (%)		296 (59%)
Living situation		
Cohabitating, *n* (%)		251 (50%)
Group allocation		
Intervention group, *n* (%)		336 (67%)
Cognitive status (MMSE), *M* (SD) scale: 0–30		22.2 (5.4)
Functional status (B-ADL), *M* (SD) scale: 1–10		7.3 (2.6)
Charlson comorbidity index, *M* (SD) scale: 0–37		3.5 (2.3)
Social support (total), *M* (SD) scale: 1–5		4.0 (0.7)
Emotional support, *M* (SD)		4.1 (0.8)
Practical support, *M* (SD)		3.9 (0.8)
Social integration, *M* (SD)		3.9 (0.7)

### Survival analyses for clinical variables and social support

Table 2 presents the results of the Cox regression analysis with social support (overall) and socio-demographic and clinical variables as predictors of life expectancy in PwD (Models 1.1 and 1.2). When social support was the sole predictor in the model, it significantly predicted life expectancy (Model 1.1). After controlling for known socio-demographic variables such as age, sex and clinical variables like cognitive status, functional status and comorbidity burden, social support remained a stable predictor of life expectancy. Each one-point increase in social support was associated with a 22% reduction in mortality risk. Furthermore, the well-known demographic and clinical variables also predicted life expectancy. Higher age significantly increased mortality risk, with an 8% increase in mortality risk per year of age. Sex significantly influenced life expectancy, with women having a 36% lower mortality risk than men. Cognitive status was a significant predictor of life expectancy, with each additional point on the MMSE scale reducing the mortality risk by 4%. Functional status also predicted the risk of mortality, with each additional point in functional ability reducing the risk by 9%. Comorbidities did not have a significant influence on life expectancy in our sample, nor did the living situation or group allocation. Sensitivity analyses based on the original, unimputed data yielded nearly identical results as the analyses based on the imputed data (see Tables S1 and S2 in the Supplement), supporting the robustness of the findings.

**Table 2:** Social support, socio-demographic and clinical variables as predictors of mortality

			95% CI				
		HR	Lower	Upper	*z*	*p*	
Model 1.1	Social support (overall) *scale: 1–5*	0.70	0.57	0.84	−3.68	<0.001	***
Model 1.2 (combined model)	Age *in years*	1.08	1.05	1.11	5.71	<0.001	***
	Sex *female versus male (ref.)*	0.64	0.48	0.85	−3.06	0.002	**
	Living situation *cohabitating versus living alone (ref.)*	1.00	0.75	1.33	−0.01	0.990	
	Group allocation *intervention versus CAU (ref.)*	0.81	0.60	1.09	−1.38	0.167	
	Cognitive status (MMSE) *scale: 0–30*	0.96	0.93	0.98	−3.11	0.002	**
	Functional status (B-ADL) *scale: 1–10*	0.91	0.86	0.97	−3.09	0.002	**
	Charlson comorbidity index *scale: 0–37*	1.03	0.97	1.10	1.08	0.282	
	Social support (overall) *scale: 1–5*	0.78	0.63	0.98	−2.18	0.029	*

All results are pooled estimates based on 20 imputed datasets.

***p < .001, **p < .01, *p < .05.

**Figure 1:**
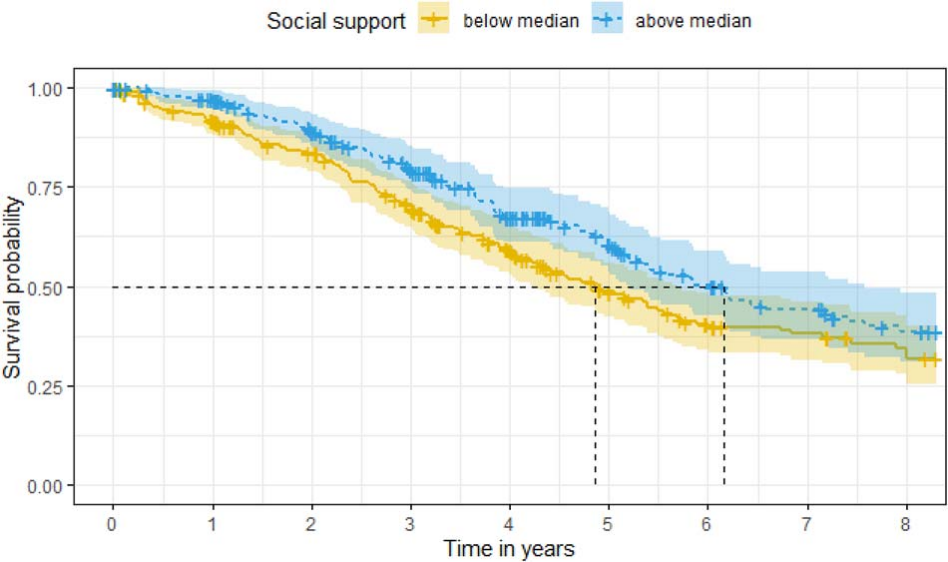
Schematic illustration of the effect of social support on mortality. *Note.* Kaplan–Meier curve as schematic illustration of the effect of social support on the survival probability without adjustment for socio-demographic and clinical variables. Individuals with dementia were divided into two groups with lower and higher social support using a median split. The analysis was conducted based on 20 imputed datasets. Crosses represent censored observations.

For illustrative purposes, participants were divided into two groups based on their levels of social support using a median split. Figure 1 displays the unadjusted survival probabilities for the two groups. The group with higher perceived social support had a higher survival probability than the group with lower perceived social support. The unadjusted median survival time in the group with higher social support was more than 1 year longer (6.2 years, 95% CI [5.2, 7.6]) than in the group with lower perceived social support (4.9 years, 95% CI [4.2, 5.7]).

### Survival analyses for subdimensions of social support

Separate Cox regressions were calculated for the different dimensions of social support. Table 3 presents the results for emotional support (Model 2.1, 3.1), practical support (Model 2.2, 3.2) and social integration (Model 2.3, 3.3). Only the dimension of emotional support significantly predicted life expectancy after adjustment for socio-demographic and clinical variables, the subdimensions practical support and social integration did not.

**Table 3:** Subdimensions of social support as predictors of mortality.

			95% CI				
		HR	Lower	Upper	*z*	*p*	
Model 2.1	Emotional support *scale: 1–5*	0.75	0.64	0.88	−3.41	<0.001	***
Model 2.2	Practical support *scale: 1–5*	0.79	0.67	0.93	−2.89	0.004	**
Model 2.3	Social integration *scale: 1–5*	0.77	0.64	0.92	−2.86	0.004	**
Model 3.1 (combined model)	Age *in years*	1.08	1.05	1.11	5.72	<0.001	***
	Sex *female versus male (ref.)*	0.65	0.49	0.87	−2.91	0.003	**
	Living situation *cohabitating versus living alone (ref.)*	0.97	0.73	1.30	−0.18	0.861	
	Group allocation *intervention versus CAU (ref.)*	0.81	0.60	1.09	−1.41	0.160	
	Cognitive status (MMSE) *scale: 0–30*	0.96	0.93	0.98	−3.04	0.002	**
	Functional status (B-ADL) *scale: 1–10*	0.91	0.85	0.96	−3.39	<0.001	***
	Charlson comorbidity index *scale: 0–37*	1.04	0.98	1.10	1.17	0.243	
	Emotional support *scale: 1–5*	0.78	0.65	0.94	−2.66	0.008	**
Model 3.2 (combined model)	Age *in years*	1.08	1.05	1.11	5.67	<0.001	***
	Sex *female versus male (ref.)*	0.63	0.47	0.84	−3.14	0.002	**
	Living situation *cohabitating versus living alone (ref.)*	1.05	0.79	1.39	0.34	0.731	
	Group allocation *intervention versus CAU (ref.)*	0.83	0.62	1.11	−1.24	0.214	
	Cognitive status (MMSE) *scale: 0–30*	0.95	0.93	0.98	−3.20	0.001	**
	Functional status (B-ADL) *scale: 1–10*	0.91	0.86	0.96	−3.20	0.001	**
	Charlson comorbidity index *scale: 0–37*	1.03	0.97	1.09	0.96	0.338	
	Practical support *scale: 1–5*	0.89	0.75	1.07	−1.23	0.219	
Model 3.3 (combined model)	Age *in years*	1.08	1.05	1.11	5.76	<0.001	***
	Sex *female versus male (ref.)*	0.63	0.47	0.84	−3.15	0.002	**
	Living situation *cohabitating versus living alone (ref.)*	1.04	0.78	1.37	0.25	0.806	
	Group allocation *intervention versus CAU (ref.)*	0.84	0.63	1.12	−1.18	0.238	
	Cognitive status (MMSE) *scale: 0–30*	0.95	0.93	0.98	−3.25	0.001	**
	Functional status (B-ADL) *scale: 1–10*	0.91	0.86	0.97	−3.01	0.003	***
	Charlson comorbidity index *scale: 0–37*	1.03	0.97	1.09	1.05	0.293	
	Social integration *scale: 1–5*	0.90	0.74	1.10	−1.02	0.307	

All results are pooled estimates based on 20 imputed datasets.

***p < 0.001, **p < 0.01, *p < 0.05.

## Discussion

The social environment is increasingly recognised as significant in dementia research, with the COVID-19 pandemic underscoring the crucial role it plays for the well-being and symptom burden of individuals with dementia [10, 12]. The aim of our study was to investigate the role of social support on the life expectancy in PwD over a time period of up to 8 years using data from community-dwelling PwD in Germany. Specifically, we focused on perceived social support, which encompasses the ‘perception or experience of being cared for, esteemed, and part of a mutually supportive social network’ [17]. We demonstrate that not only known demographic and clinical factors, such as age, sex or severity of dementia predicted life expectancy but also support from the social environment. When considering social support without accounting for other predictors of life expectancy, the median survival time of individuals with greater perceived social support was 1 year higher than the median survival time of those with lower social support.

### Potential pathways between social support and life expectancy

How does social support affect life expectancy in PwD? Two fundamental assumptions regarding the mechanisms of social support are commonly discussed. Firstly, the ‘direct-effect model’ posits that meeting the fundamental human need for connection and belonging directly contributes to well-being and overall health [35, 36]. Secondly, the ‘buffer-effect model’ proposes that social support positively influences the processing of stressors and buffers the negative effects of stress on well-being and health [35, 36]. In this context, dementia as a chronic neurodegenerative syndrome can be considered a significant stressor, where social support serves as a valuable resource for alleviating the adverse effects of the condition.

Moreover, on a neurobiological level, three primary pathways have been studied through which social support influences well-being and health: the autonomic nervous system (including the cardiovascular system and heart rate variability), the neuroendocrine system (such as the hypothalamic–pituitary–adrenal axis and oxytocin) and the immune system [17, 20]. Evidence from these and other studies suggest that social support can reduce adverse activation patterns of these systems. This could explain why dementia patients with more social support have a longer life expectancy.

### The special role of emotional support

The study explored which subdimensions of social support predicted life expectancy in PwD. Controlling for previously reported socio-demographic and clinical variables, only the effect of emotional support was found to be significant. Emotional support refers to the experience of receiving ‘love, caring, sympathy, understanding, and a sense of value from others’ [15, 37]. Its assessment involved items that asked if the PwD know someone who accepts them as they are, who shares joy and sorrow with them and with whom they can openly share their thoughts and feelings. In contrast, there was no significant effect for the dimensions of practical support, involving assistance with daily tasks, or social integration, which relates to a sense of belonging to a social network.

Indeed, emotional support seems to play a special role in the well-being of individuals. In the context of the buffer-effect model, it is assumed that particularly emotional support can function as a buffer for stress during major life events [36, 38]. Our results provide initial evidence that this might also be the case with a life-altering event such as the onset of and living with a neurodegenerative disease such as dementia. The literature also shows that a lack of emotional support is an important predictor of loneliness [39], which in turn is associated with a shorter life expectancy [40].

### Strengthening social and emotional support is challenging

Our results suggest that a lack of social support, particularly emotional support, is a risk factor for mortality in PwD, beyond clinical factors. In the past, the focus was more on the nursing and physical needs of PwD, but our study provides further evidence that psychosocial needs should also be more strongly emphasised.

A lack of emotional and social support is a complex, multifaceted issue. To our knowledge, specific measures and interventions aimed at directly promoting emotional support are scarce and have not been implemented in routine care. Additionally, it greatly depends on the individual circumstances as to which approaches may be suitable for enhancing emotional support for a person. An important starting point would be to identify situations where there is a significant change in emotional support for an older person, for instance, by general practitioners who often serve as an important point of contact for PwD. A decline in social and emotional support often occurs during critical life events, such as the loss of a spouse, other close family members or close friends [41, 42]. Possible offerings to mitigate the loss of emotional support could include grief counselling, training for relatives if other close family members are present, as well as services from social care, such as social prescribing [43, 44]. However, these require an attentive environment, precise identification of emotional support needs and tailored recommendations for each individual.

### Limitations

One limitation is the lack of a validated dementia diagnosis for inclusion in the study. The DelpHi-MV trial was not a diagnostic trial and inclusion of PwD was based on a screening instrument applied by the patients’ general practitioner. A state-of-the-art diagnostic procedure was not used. However, the DemTect was designed for this specific purpose and is widely used in routine care in Germany [45]. The close integration of the study in the routine care setting adds to the external validity of its results. Another limitation arises from the substantial attrition rate throughout the long observation period, which is attributable to the age and health status of the study participants.

Furthermore, the questionnaire used to assess perceived social support was not explicitly validated for individuals with cognitive impairment. There are, however, indications for the questionnaire’s validity in this sample. Validation was conducted not only in population-representative samples but also in samples of individuals with mental health diagnoses and hospitalised orthopaedic patients [26]. Furthermore, the questionnaire has been used in various geriatric samples [46, 47], including individuals with dementia [48, 49]. An advantage of the questionnaire is that it was developed and validated in Germany, making it culturally appropriate to assess social support in the German context.

Finally, in our study, we did not find an influence of the Charlson Comorbidity Index on life expectancy. However, this index was originally developed to predict life expectancy in hospitalised patients and may not have been sensitive enough in our sample of community-dwelling PwD. Many of the diagnoses that are heavily weighted in the Charlson Comorbidity Index (e.g. metastatic solid tumour, moderate or severe liver disease, HIV/AIDS) were either not present or very rare in our sample.

## Conclusion

Our study provides novel evidence that lower perceived support from the social environment is a risk factor for mortality in PwD—and this extends beyond known clinical risk factors like dementia severity. Specifically, emotional support appears to play a crucial role in the well-being and life expectancy of PwD. Our study further highlights the importance of addressing not just the physical and healthcare needs of PwD but also their psychosocial needs.

## Supplementary Material

aa-23-1796-File002_afae044
