# Supplementary material for: What influences life expectancy in people with dementia? Social support as an emerging protective factor
Source: Age Ageing. 2024 Mar 15;53(3):afae044. doi: 10.1093/ageing/afae044 (PMC10945357; doi:10.1093/ageing/afae044)

# Supplementary Material to “What Influences Life Expectancy in People with Dementia? Social Support as an Emerging Protective Factor”

**eTable 1.** Social Support, Sociodemographic and Clinical Variables as Predictors of Mortality (unimputed data).

|  |  |  | 95% CI | |  |  |  |
| --- | --- | --- | --- | --- | --- | --- | --- |
|  |  | HR | lower | upper | *z* | *p* |  |
| Model 1.1 | Social support (overall)  *scale: 1 – 5* | 0.71 | 0.58 | 0.87 | -3.35 | <0.001 | *** |
| Model 1.2 | Age  *in years* | 1.08 | 1.05 | 1.11 | 5.03 | <0.001 | *** |
| (combined model) | Sex  *female vs. male (ref.)* | 0.57 | 0.42 | 0.78 | -3.52 | <0.001 | ** |
|  | Living situation  *cohabitating vs. living alone (ref.)* | 1.01 | 0.74 | 1.36 | -0.04 | 0.971 |  |
|  | Group allocation  *intervention vs. CAU (ref.)* | 0.79 | 0.57 | 1.08 | -1.47 | 0.142 |  |
|  | Cognitive status (MMSE)  *scale: 0 – 30* | 0.96 | 0.93 | 0.99 | -2.61 | 0.009 | ** |
|  | Functional status (B-ADL)  *scale: 1 – 10* | 0.88 | 0.83 | 0.94 | -3.91 | <0.001 | ** |
|  | Charlson comorbidity index  *scale: 0 – 37* | 1.03 | 0.97 | 1.10 | 0.99 | 0.323 |  |
|  | Social support (overall)  *scale: 1 – 5* | 0.79 | 0.63 | 0.98 | -2.12 | 0.034 | * |

*Note*. HR = Hazard Ratio, CI = Confidence Intervals, CAU = Care as Usual. *** *p* < .001, ** *p* < .01, * *p* < .05.

**eTable 2.** Subdimensions of Social Support as Predictors of Mortality (unimputed data).

|  |  |  | 95% CI | |  |  |  |
| --- | --- | --- | --- | --- | --- | --- | --- |
|  |  | HR | lower | upper | *z* | *p* |  |
| Model 2.1 | Emotional Support  *scale: 1 – 5* | 0.76 | 0.64 | 0.90 | -3.17 | 0.002 | *** |
| Model 2.2 | Practical Support  *scale: 1 – 5* | 0.80 | 0.68 | 0.94 | -2.64 | 0.008 | *** |
| Model 2.3 | Social Integration  *scale: 1 – 5* | 0.78 | 0.65 | 0.94 | -2.66 | 0.008 | *** |
| Model 3.1  (combined model) | Age  *in years* | 1.08 | 1.05 | 1.11 | 5.00 | <0.001 | **** |
|  | Sex  *female vs. male (ref.)* | 0.60 | 0.44 | 0.82 | -3.25 | 0.001 | *** |
|  | Living situation  *cohabitating vs. living alone (ref.)* | 0.99 | 0.73 | 1.34 | -0.07 | 0.942 |  |
|  | Group allocation  *intervention vs. CAU (ref.)* | 0.77 | 0.57 | 1.06 | -1.61 | 0.107 |  |
|  | Cognitive status  *scale: 0 – 30* | 0.95 | 0.93 | 0.99 | -2.90 | 0.004 | *** |
|  | Functional status (B-ADL)  *scale: 1 – 10* | 0.88 | 0.83 | 0.94 | -4.09 | <0.001 | *** |
|  | Charlson comorbidity index  *scale: 0 – 37* | 1.03 | 0.97 | 1.10 | 1.05 | 0.295 |  |
|  | Emotional support  *scale: 1 – 5* | 0.78 | 0.65 | 0.94 | -2.67 | 0.008 | *** |
| Model 3.2 (combined model) | Age  *in years* | 1.08 | 1.05 | 1.11 | 5.67 | <0.001 | **** |
|  | Sex  *female vs. male (ref.)* | 0.59 | 0.43 | 0.80 | -3.14 | 0.001 | *** |
|  | Living situation  *cohabitating vs. living alone (ref.)* | 1.07 | 0.80 | 1.44 | 0.34 | 0.635 |  |
|  | Group allocation  *intervention vs. CAU (ref.)* | 0.80 | 0.59 | 1.10 | -1.24 | 0.166 |  |
|  | Cognitive status (MMSE)  *scale: 0 – 30* | 0.95 | 0.92 | 0.98 | -3.20 | 0.003 | *** |
|  | Functional status (B-ADL)  *scale: 1 – 10* | 0.89 | 0.83 | 0.94 | -3.20 | <0.001 | *** |
|  | Charlson comorbidity index  *scale: 0 – 37* | 1.03 | 0.97 | 1.09 | 0.96 | 0.407 |  |
|  | Practical support  *scale: 1 – 5* | 0.91 | 0.76 | 1.09 | -1.23 | 0.309 |  |
| Model 3.3  (combined model) | Age  *in years* | 1.08 | 1.05 | 1.11 | 5.02 | <0.001 | **** |
|  | Sex  *female vs. male (ref.)* | 0.58 | 0.43 | 0.79 | -3.44 | 0.001 | *** |
|  | Living situation  *cohabitating vs. living alone (ref.)* | 1.05 | 0.78 | 1.42 | 0.35 | 0.727 |  |
|  | Group allocation  *intervention vs. CAU (ref.)* | 0.81 | 0.59 | 1.10 | -1.35 | 0.177 |  |
|  | Cognitive status (MMSE)  *scale: 0 – 30* | 0.95 | 0.92 | 0.98 | -3.03 | 0.002 | **** |
|  | Functional status (B-ADL)  *scale: 1 – 10* | 0.89 | 0.84 | 0.95 | -3.59 | <0.001 | *** |
|  | Charlson comorbidity index  *scale: 0 – 37* | 1.03 | 0.97 | 1.09 | 0.92 | 0.359 |  |
|  | Social integration  *scale: 1 – 5* | 0.89 | 0.73 | 1.10 | -1.08 | 0.281 |  |

*Note*. HR = Hazard Ratio, CI = Confidence Intervals, CAU = Care as Usual. *** *p* < .001, ** *p* < .01, * *p* < .05.

**eFigure 1.** Participant flow over eight years in the DelpHi-MV trial.


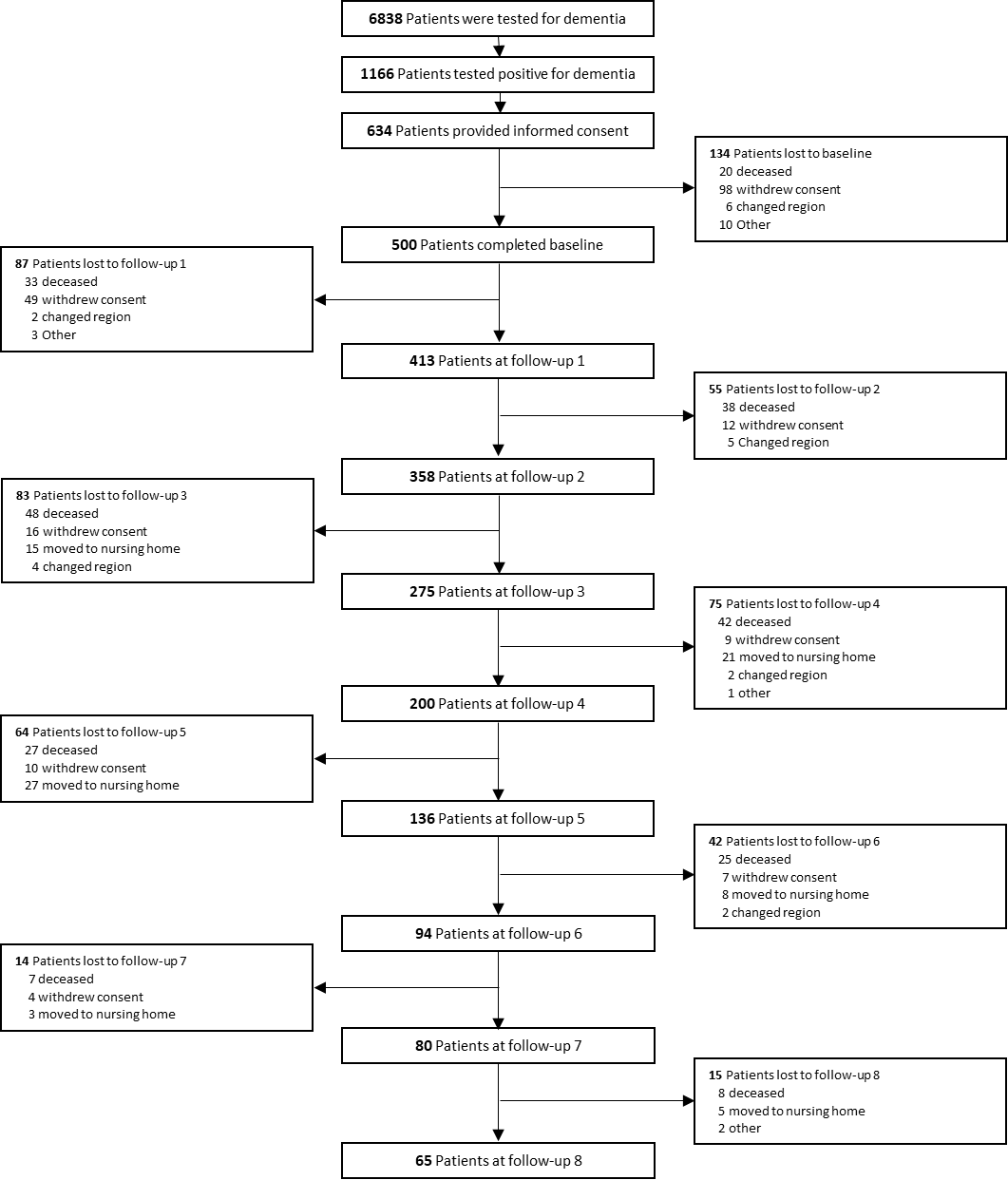

Supplement: aa-23-1796-File002_afae044 [file aa-23-1796-file002_afae044.docx]
